# Silica Aerogels as a Promising Vehicle for Effective Water Splitting for Hydrogen Production

**DOI:** 10.3390/molecules30061212

**Published:** 2025-03-08

**Authors:** Apurva Vadanagekar, Lubomir Lapcik, Libor Kvitek, Barbora Lapcikova

**Affiliations:** Department of Physical Chemistry, Faculty of Science, Palacky University in Olomouc, Listopadu 12, 771 46 Olomouc, Czech Republic; apurva.vadanagekar01@upol.cz (A.V.); libor.kvitek@upol.cz (L.K.); barbora.lapcikova@upol.cz (B.L.)

**Keywords:** silica aerogel, environmental remediation, adsorbent, hydrogen generation, dye degradation

## Abstract

This comprehensive review explores silica aerogels and their application in environmental remediation. Due to rapid growth in the consumption of energy and water resources, the purification of contaminated resources for use by humankind should be considered important. The primary objectives of this review are to assess the evolving landscape of silica aerogels, their preparation, and drying techniques, and to discuss the main findings from a wide range of empirical studies and theoretical perspectives. Based on a significant amount of research, this review provides information about aerogels’ capabilities as an adsorbent and catalyst. The analysis spans a variety of contexts for the generation of hydrogen and the degradation of the dyes employed in industry, showing better performance in environmental remediation. The implications of this review point to the need for well-informed policies, innovative synthesis strategies, and ongoing research to harness the full potential for environmental management.

## 1. Introduction

**Figure 1:**
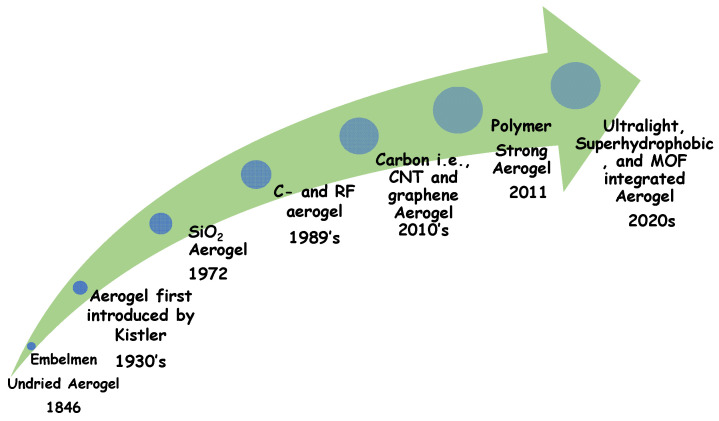
Modification of aerogel through the years.

In 1931, Steven S. Kistler was the first to introduce the term ‘aerogel’, in which the liquid/moisture is replaced with a gas, without disturbing the solid gel network. The IUPAC nomenclature defines aerogels as a “Gel comprised of a microporous solid in which the dispersed phase is a gas”. Though this statement is not accepted by most scientific researchers, they state that some aerogels show nanofibril and mesoporous structures [1]. The initial gels are produced through sol–gel reactions or a thermo-reversible gelation process. The pore diameter and the shape of the aerogel can be altered at the nano level to achieve versatile properties, like a high surface area, low density, a large pore size, and low thermal conductivity [2]. The modification of the aerogel is shown in Figure 1.

**Figure 2:**
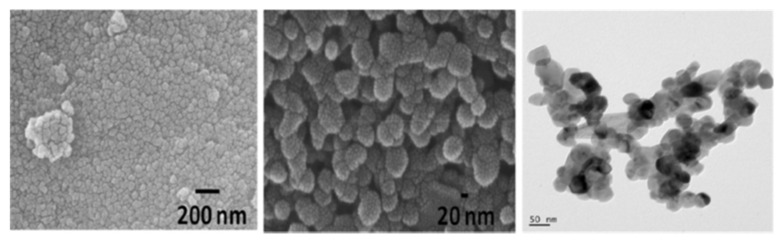
Electron microscope image of highly porous, continuous 3D network structure of aerogel layer (reprinted from S Alwin et al. [3] with permission. Copyright 2020, Spinger nature).

Aerogels have a porous, continuous network structure, as depicted in Figure 2 using electron microscopy pictures [3]. Furthermore, because of their density, which is in the middle of a liquid and gaseous state, aerogels are regarded as a new form of matter. The traditional aerogel is commonly called ‘frozen smoke’, because it possesses open cells, mesoporosity, low-density solids, and a foam-like structure [4]. They have solid particles with enduring characteristics like being ultra-lightweight and having nano-pores with cross-linking; these cross-linked pores have sizes ranging from 5 to 100 nm and an average diameter of 20 to 40 nm [5].

Solid foams are a diverse type of porous material that is formed by gas pockets getting trapped in a mold of solid. They are frequently identified by significant macoporosity and low bulk densities. The foaming method, also known as “aerogel foam’’, blends both open and ‘closed’ pore aerogels, making the foam an innovative but unusual ordered structure with open pores; the vacant pores of closed foam have a larger size and cross-linking in other possible directions along the intrinsic pores of the aerogel [6]. Hollow sphere-based silica foam composites are a potential insulation component due to their high porosity and similarity to silica aerogels, with the conductivity being considerably limited by a lighter solid phase and concentrated gas phase in the inner as well as outer pores of the hollow spheres [7]. The addition of a polymer to silica aerogels enhances the connectivity between the silica particulates, allowing them to create extra-strong C–C covalent bonds. This is because silica aerogels are composed of tiny bits of silica that are linked together by Si-O-Si bonds. Therefore, in comparison to pristine silica aerogels, this method increases the strength of reinforced silica aerogels [8]. 

The rising worldwide need for energy should be overcome, so it is essential to develop environmentally friendly energy with modern technology that eliminates reliance on fossil fuels, mitigates the negative environmental effects, and lowers costs [9]. Moreover, implementing clean energy technology in our daily routines can lead to a reduction in greenhouse gas emissions and waste. The advanced conversion and preservation of energy systems emerged through the utilization of rechargeable lithium batteries, fuel and solar cells, and electrochemical and photocatalytic water splitting [3]. Aerogels are a distinctive category of porous materials, characterized by their exceptionally low relative density, attributable to their high porosity and considerably smaller pore size, often below 100 nm, compared to foams [10]. Aerogels, characterized by their large porosity (often above 80 vol%) and specific surface area (about 100–1000 m^2^/g), have been synthesized in diverse compositions, and their applications have been investigated, including their thermal insulation, energy storage, catalytic features, environmental remediation, and aerospace applications [11,12].

**Figure 3:**
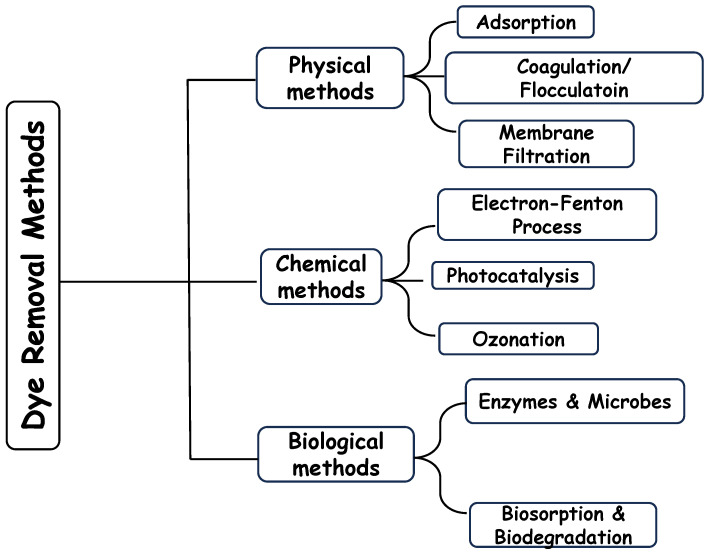
The flow chart represents different methods for the removal of dyes.

The dye is also known as a coloring agent with organic compounds, which are attached to the surface of the clothing (fabric) to add color. They have acidic properties with water solubility realizing bright or light colors in the water [13]. Natural resources are the primary source of dyes, which are not subjected to physical or chemical processing. Each year, 800,000 tons of dye effluents is produced by the global textile sector. About 75% of dyes on the market are synthetic dyes, and significant levels of untreated synthetic dyes are frequently found in dyeing process effluents. The three types of dyes that rely on charged particles are cationic, anionic, and non-ionic [14]. Dyes have the potential to negatively impact aquatic ecosystems and inhibit the penetration of sunlight into water, thereby slowing down the process of photosynthesis. The concentration of dye in water needs to be either removed or lowered to an acceptable level prior to drainage [15]. Figure 3 shows a flow chart of the methods for the removal of dyes [13].

I. Cationic—contains dyes which are basic in nature or characteristics.
II. Anionic—reactive, acid containing, azo and direct dyes.
III. Non-ionic—dyes that distribute and remain non-ionized in water-based media.

Conventional approaches to wastewater treatment encompass a variety of techniques, including chemical methods, such as physical adsorption, physicochemical, electrochemical, biochemical, and catalytic degradation [16]. The elimination of these dyes is a crucial factor; the accumulation of a particular substance on the surface of some other phase, often solid or liquid, due to physical or chemical forces is referred to as “adsorption” [17]. The adsorption technique, considered for its simplicity and effectiveness, has garnered significant attention in recent studies, particularly in the context of adhesion dyes [18,19]. The elements that are involved in the technique of adsorption are referred to as adsorbents [17]. Mesocellular foam (MCF) silica is characterized by its three-dimensional mesoporous architecture and significant pore dimensions [20]. In comparison to mesoporous materials, mesocellular foam silica molecular sieves have a large-diameter spherical chamber and a uniform window, so they are expected to be a suitable adsorption material [21].

This review’s goal is to present current data and provide a comprehensive analysis of hydrogen production, transit, use, and storage. The present summary attempts to shed light on the many technologies used in each step of the hydrogen value chain by thoroughly examining the most recent developments in technology. The discussion in this review is intended to aid our journey to the future of neutral energy and increase awareness concerning environmentally friendly hydrogen systems. The overall aim is to fill a specific gap in the current understanding of silica aerogels in water-splitting applications by focusing on silica aerogel and the ability of silica aerogel in the production of hydrogen via catalytic or photocatalytic water splitting, which has been not discussed in recent reviews, including the detailed analysis of surface area and porosity, hydrophilicity, electronic band structure, charge carrier dynamics, stability, and metal/oxide doping effects. This approach provides a comprehensive overview of the critical parameters influencing the catalytic and photocatalytic effectiveness of silica aerogels, which has not been thoroughly addressed in recent publications.

Therefore, there is a need to develop a catalyst or photocatalyst or nanocomposite to produce hydrogen from water splitting, and, also, the removal of dyes from aqueous media is an important environmental issue from an environmental safety point of view.

## 2. Types of Aerogels

**Figure 4:**
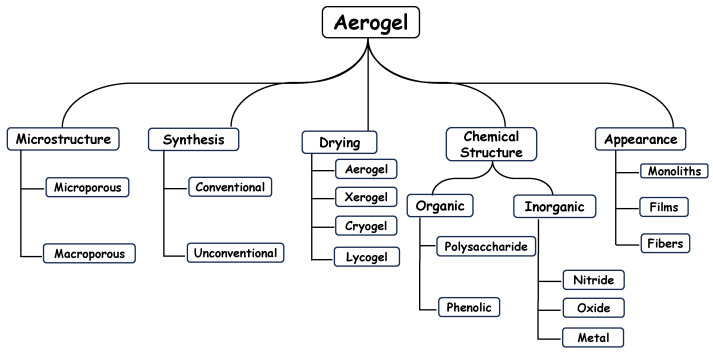
Classification of aerogel based on synthesis, drying, appearance, microstructure, as well as chemical structure.

The types of aerogels are dependable because of the synthesis, drying, appearance, microstructure, as well as the chemical structure, which can be observed in Figure 4 [22]. The aerogel, which has a pore diameter of less than 2 nm, is known as microporous, while some having 250 nm are called mesoporous and mixed porous aerogels [23,24]. The aerogels are also differentiated by the synthesis process, such as conventional and unconventional sol–gel synthesis. They can also be subdivided because of the drying methods, like supercritically dried gels are aerogels, freeze-dried gels are cryogels, ambient pressure or evaporatively dried gels are xerogels, and lycogels are produced through a process called lyophilization [1]. The aerogel is classified based on how it appears; monoliths are homogenous with ease of assembly and recyclability, and films are employed in the field of electronics, fibers in the textiles, and supercapacitors because of their gelation and effective wet spinning [25,26]. The aerogel is also distinguished based on the organic aerogels containing polyol, phenolic, protein, polysaccharide, and carbon-based aerogels, while inorganic aerogels, i.e., from oxide or metal oxides, include manganese, stannic, titania, and alumina [1,27]. From carbides like silicon and carbon aerogel, nitride has carbon nitride and aluminum nitride aerogels. The metal aerogel with nanoparticles and nanofoams is made of iron, gold, and silver [1].

### 2.1. Chemistry of Sol–Gel

Flory, in 1974, classified gels into four categories: disordered particle gels, covalent polymer networks, networks of physically aggregated polymers, and organized, lamellar gels. Then, in 1996, Kakihana’s classification of the many gel types obtained via sol–gel chemistry—a method used to generate inorganic solids—became more useful. Because sols and gels are so diverse, materials based on them can be categorized into several kinds [2].

**Figure 5:**
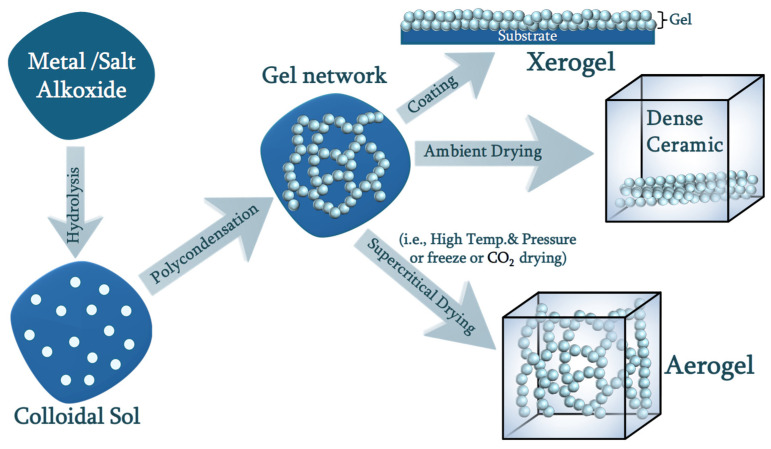
Typical schematic methodology representation for the sol–gel formation based on drying of gel.

The aerogel is synthesized through the sol–gel technique at low temperatures of no more than 100 °C, a bottom-up approach in a liquid form. In this process, the formation of the sol from different precursors like alkoxide, hydroxide, and salt is employed [28]. The sol is prepared by using methanol and ethanol as solvents. Figure 5 represents the formation of sol–gel and the gel being distinguished in the drying process. The inorganic aerogel is prepared by the transition of sol–gel, in which two steps are involved: (a) sol—the metal or the other precursor forms a liquid colloidal suspension ranging from 1 to 1000 nm; (b) gel—formed by a cohesive 3D network between interlinked atoms. The typical polycondensation reaction involves particles extending to their proper size during condensation, while dispersion happens when big particles are shrunk to colloidal proportions. Sols are produced more frequently when the rate of nucleation is high and the rate of crystal growth is low. The diameter of the inorganic clusters can vary from 10 to 200 Å, contingent upon the degree of cross-linking and the formation process. These clusters can be colloidal or polymeric in nature [29]. Flory’s classification suggests that gels can be considered/assumed to be particulate or polymeric. The solid network has a polymeric chain with a firm backbone with particles of <1.5 nm size, which is not possible to see through the human eye but with the help of electron microscopy technology [2].

### 2.2. Synthesis of Silica Aerogel

The primary precursor for sol in the synthesis of silica aerogel is silicon alkoxides. There are precursors, like tetraethoxysilane (Si(OC_2_H_5_)_4_), tetramethoxysilane (Si(OCH_3_)_4_), and polyethoxy disiloxane SiO_n_(OC_2_H_5_)_4-2n_, for a synthesis silica-based aerogel [24]. The high-quality transparent form of aerogel can be prepared from SiO_n_(OC_2_H_5_)_4-2n_, which is considered to be in demand. The (Si(OCH_3_)_4_) and SiO_n_(OC_2_H_5_)_4-2n_ show lower thermal conductivity than (Si(OC_2_H_5_)_4_), which are monolithic aerogels in the parameters of the conductivity spectrum [30]. The formation of sol leads to hydrolysis and polycondensation, where the molecule of the precursor produces a siloxane (Si-O-Si) bridge, resulting in a nanostructured solid network. The process of hydrolyzing silicon alkoxide (Si(OR)_4_) yields silica sol. The silicone alkoxide reactant (Si(OR)_4_) and the OH-group react, eliminating the alkoxide group and producing silanol as a byproduct. Alcohol is created as a byproduct of the reaction between the H^+^ ion and the alkoxide group. In an acidic atmosphere, the speed of reaction accelerates [31,32,33]. After gelation, aging, and drying of the gel, aerogel formation is involved. Table 1 represents the various synthesis techniques of aerogels and the different precursor aerogels with their advantages and disadvantages.

## 3. Aging

The pores in the gel network contain inactive bonds between Si-OH and Si-OR and trapped shrunk monomers. To improve the strength inside the gel network and secondary particles with the siloxane bonds, the ‘aging’ process is a critical step [8]. Accordingly, there might be two mechanisms that can disturb the structure of the gel: (i) development in the region of the Si-O-Si because silica suspended from the surface of particles is reprecipitated over the bridge between secondary particles; (ii) Ostwald ripening mechanism, with the disintegration of smaller particles and residue formation over the larger particles. If aging is increased, the aerogel shows a decrease in bulk density and contraction, different to gels dried at room temperature (ambient dried has a larger pore size and volume). In aging techniques, ethanol–siloxane blends are frequently employed. After the gel has matured, it must be wiped with ethanol to remove any remaining water from the pores [4,30].

## 4. Drying

The most important and last step for the formation of aerogels is the drying of the gel. In this technique, the gel is dried to remove the remaining moisture from the gel network or any extra solvent, which will be evaporated while drying, keeping the gel network intact or without breaking it. There are four main drying methods: (a) Evaporation—the process of vaporization of the liquid in the gels. (b) Ambient pressure—the drying of the gels initially occurs via evaporation and further by high-temperature drying but at ambient pressure [34]. (c) The supercritical drying technique contains a system where the gel is in a supercritical state with no liquid–gas interface, and then the fluid is gradually released into the atmosphere. As there is no capillary action in this final step of drying, the absence of a liquid–vapor interface is observed. After the drying process, the gel network retains its highly porous nature. (d) The freeze-drying technique is known, in which the solvent inside the pores needs to cross the liquid–solid and solid–gas equilibrium curves [35]. (e) Vacuum drying is used for wet gels, which enhance the high surface area and create large pore sizes in the gels. (f) Microwave drying is employed for structures with mesoporous and microporous 3D cross-linked gels, which also helps to reduce the drying time [24].

### 4.1. Evaporation

The elimination or vaporization of the liquid content from the wet gels and obtaining a thin layer of gel is reported to form Xerogels. Xerogels are primarily characterized by their dense and disintegrated materials, exhibiting a collapsed structure. The primary contributors to this structure include evaporative drying as well as the precipitation of residual reactive groups, in that hydroxyl (-OH) and alkoxy (R-O) groups on the silica are sufficiently proximate to facilitate a reaction because of compression, leading to an increase in siloxane bond formation [11].

### 4.2. Ambient Pressure

The risks and high costs associated with the freeze-drying and supercritical drying methods make them unsuitable for the industrial manufacturing of aerogels [24]. Nonetheless, ambient pressure drying is a relatively straightforward and cost-effective method for producing aerogels on a large scale [4]. Two approaches, however, can be used to create aerogels with minimal volume shrinkage and strong mechanical stability under APD circumstances, hence reducing the drying stress. The capillary stress is lowered during APD. The first tactic uses a low-surface-tension solvent during the solvent exchange. Low-surface tension solvents, such as hexane and acetone, have been shown in several experiments to be effective in lowering capillary stress during APD. Increasing the gel framework’s strength to sustain high capillary stress during APD is an alternative tactic. The APD technique has been utilized by numerous researchers to create various types of aerogels, such as silica-based aerogels, polymer-reinforced silica aerogels, polymer-modified silica aerogels, and radiofrequency aerogels [2]. Ambient pressure drying, also known as evaporation, is the removal of fluid or moisture inside the pore of the gel, where the pressure of a closely packed container surpasses the liquid–gas equilibrium curve [35].

### 4.3. Supercritical Drying

**Figure 6:**
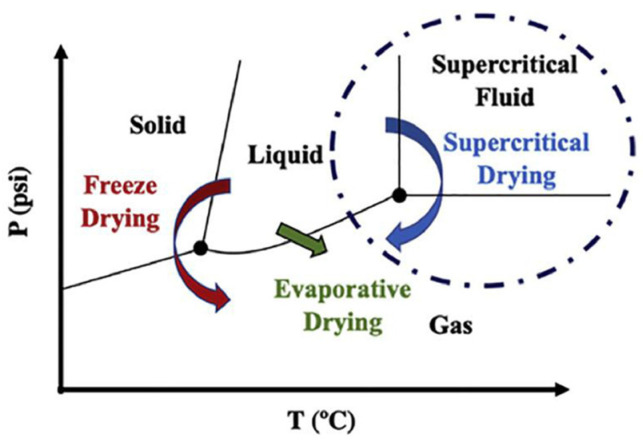
Phase diagram of liquid to convert into gel network (reprinted from Suhail Ahmad, Sameer Ahmad, Javed Nabibaksha Sheikh [11] with permission. Copyright 2023, Elsevier).

The first to introduce the supercritical drying of gels was Kistler in the 1930s [36]. The supercritical drying technique contains a system where the gel is in the supercritical state with no liquid–gas interface; then, the fluid is gradually released into the atmosphere. As there is no capillary action in this final step of drying, a liquid–vapor interface is observed. There are two common approaches used to pursue supercritical drying techniques. The primary method, referred to as hot drying, involves supercritical drying about the pore fluid. Generally, temperatures of around 250 °C and pressures of approximately 80 bar must be maintained for alcohols (methanol or ethanol) and other organic liquids (such as acetone or acetonitrile) [37]. However, the supercritical drying method requires high pressure and temperature, which raises safety concerns. A reduction in the surface area of the gel network occurs due to rearrangement reactions under these conditions. The supercritical drying mechanism of gel is shown in Figure 6. Furthermore, a different supercritical drying method with low-temperature liquid carbon dioxide (CO_2_) was introduced to avoid these problems. The carbon dioxide in solvent form has a correspondingly low critical point. The maintenance of the porous network and structural interchange are slight in the case of CO_2_ supercritical drying. However, this method has the disadvantage of being prolonged because of the change in pore liquid with liquid CO_2_, and the duration is still long due to the subsequent solvent interchange caused by the mixture condition [3].

### 4.4. Freeze-Drying

**Figure 7:**
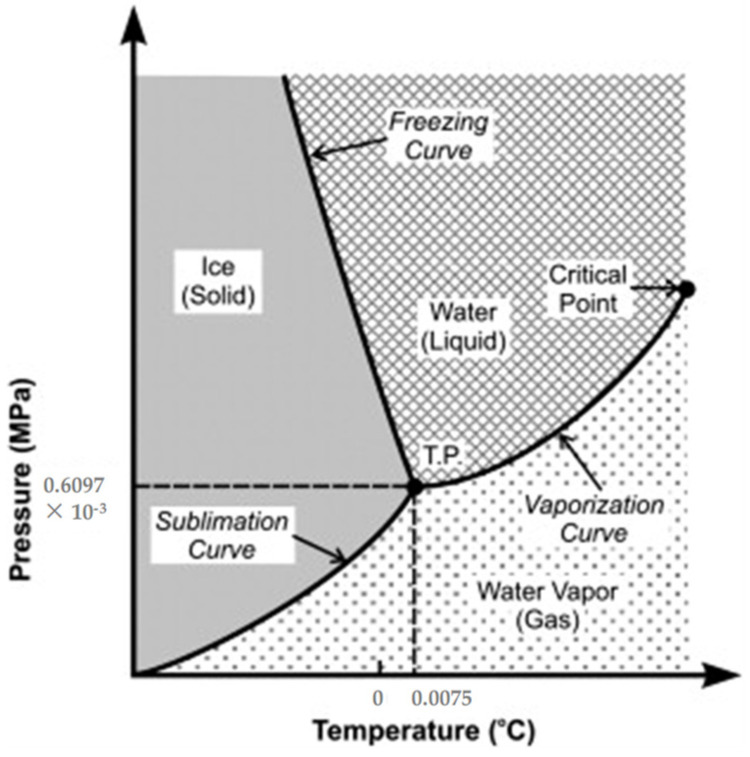
Phase diagram for freeze-drying of water (reprinted from Robert Ettema, Gokhan Kirkil, Steven Daly [40] with permission. Copyright 2009, Elsevier).

The production of porous materials can also be achieved via the straightforward, affordable, and environmentally benign process of freeze-drying, which can occasionally take longer than supercritical drying [38]. The foundation of the freeze-drying method is the dissolution of the frozen solvent, which stops defrosting upon the removal of the liquid component without reducing the solid to the vapor state [39]. The conditions under which the gelation process and freeze-drying occur have been established through advances in research on the lyophilization of gels and slurries and freeze-drying. The lyophilization process involves three steps: (i) drying of solvent with exchange in solvent, (ii) freezing, (iii) vacuum atmosphere with sublimation. Freeze drying is commonly employed to synthesize hydrogels. The gel chilling rate, the liquid phase arrangement, the water content, the solution’s viscosity, and the temperature at which it freezes determine the final configuration of the porous structure. The freeze-drying technique is known, in which the solvent inside the pores needs to cross the liquid–solid and solid–gas equilibrium curves. A freeze-drying phase diagram of water is presented in Figure 7. Some disadvantages of this approach include the difficulty in creating monoliths and the requirement to switch out solvents while avoiding the production of pores with a micrometer size of 10 μm. The arrangement of the aerogel depends on the drying technique, and which of the methods to use is also interdependent on the chemical and the relative utilization of the dried material. Therefore, all the three techniques have their disadvantages and restrictions [2].

### 4.5. Vacuum Drying

An additional drying method was introduced to obtain gels from wet gels with elevated surface area, substantial pore size, and decreased drying duration [24]. The properties of open-cell and closed-cell foams are shown in Table 2.

## 5. Properties of the Silica Aerogel

The properties or characteristics of the silica aerogel differ depending on the synthesis procedure. The physical property of the aerogel shows great consequence because of the chemical composition presented in Table 3, along with values and comments.

### 5.1. Transparency Property

**Figure 8:**
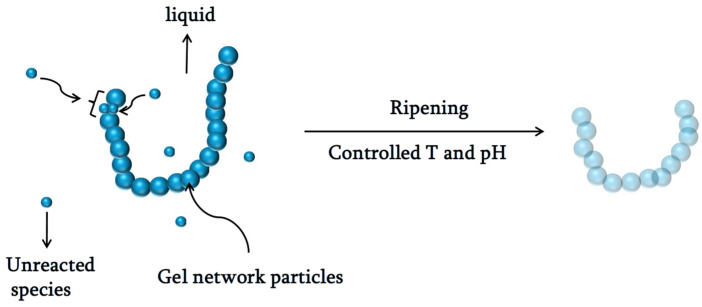
The process of Oswald ripening.

Aerogels’ pores are often too tiny to scatter visible light, but, occasionally, some of them do. The cloudy visual is caused by light being scattered by the larger pores in the aerogel [41]. The brittle nature of aerogels is a consequence of their significant porosity and the limited connection of individual colloidal particles, serving as the sole disadvantage to their utilization. This makes it difficult to handle the produced aerogels despite damaging them or dry wet gels. The percentage of trifunctional species added to the tetrafunctional monomer should be limited to between 30 and 40% to produce transparent and flexible aerogels. The aerogel structure or transparency, as well as the particle size and the pH of the solution, can be managed by changing conditions in the precursor solution of polymerization, the molar ratio of the constituent materials used to prepare the alcogel, the precursor for the aerogel, and the dimensions in the aging, particularly the process of Oswald ripening (Figure 8) or coarsening [42].

### 5.2. Thermal Property

The replacement for insulating material like polystyrene and polyurethane can be carried out by silica aerogel or blankets, as they have low heat conductivity for their inorganic characteristics. There are also different reasons for considering aerogels, which can be, for thermal insulation materials, less than half of the thickness required for aerogel-based thermal insulation materials. The inorganic-based materials or polymeric foams are less costly than the aerogel, which is 8–9-times higher. Because of the amorphous nature of the aerogels, they are considered less harmless to humans and the environment [27]. The thermal conductivity of the aerogel can be calculated.

K = 16σn^2^T^3^ξ^−1^

n = refraction index, σ = Stefan Boltzmann constant, ξ = ρ_be_ coefficient of spectral attenuation, T = temperature (K).

### 5.3. Mechanical Property

**Figure 9:**
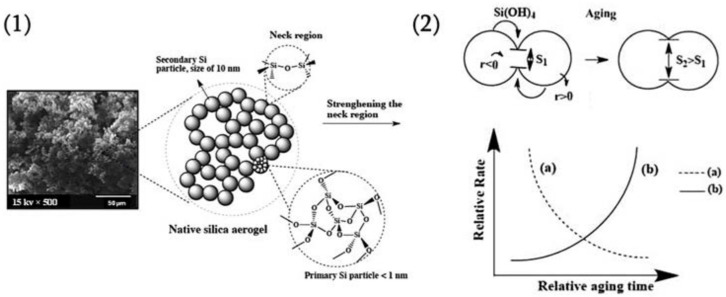
Traditional SEM image of Si aerogels alongside a schematic depiction of primary and secondary silica particles (left); (**1**) neck development process of secondary silica particles and (**2**) comparative aging rate as a function of time for two mechanisms (a, b) (reprinted from S. Ahmad et al., [11] with permission. Copyright 2023, Elsevier).

The mechanical properties of clean silica aerogels are intricately correlated with their overall density and membrane density. The skeleton arrangement of silica aerogels resembles a necklace of pearls and is present in cellular solids. From the Figure 9, it can be considered that interparticle regions have weak points in the structure. Even though the pure silica aerogels are resilient enough to bear, some of the practical applications with mechanical quality remain the same to keep them intact and monolithic. Although aerogels are naturally brittle, they can become more durable with the right age and heat treatments [33]. Silica aerogels with densities less than 0.090 g/cm^3^ cannot be considered brittle; instead, they undergo an irreversible, plastic form change upon compression, and their Young’s modulus (E) is low. Even though silica aerogels have a high Young’s modulus, they become brittle at 0.150 g/cm^3^. The average density of most conventional silica aerogels is approximately 0.120 g/cm^3^ [11]. The aging process of wet gels increases the strength of the resulting silica aerogel by causing silica to dissolve and repeated precipitation at the exterior of interparticle necks, resulting in mechanically significant inorganic connectivity. This procedure will result in an increase in the elasticity of modulus of about two aspects. However, it is evident that, regardless of the end, the strengthening component is still silica, which is a brittle substance with a low tensile strength [8].

Aerogel manufacturing conveys mechanical concerns in two distinct ways: first, by producing debris of silica aerogel (powder or granulate) as a non-fully prepared outcome, which is incorporated into a framework to provide mechanical strength; second, by incorporating silica aerogel into a delicate fiber blanket, which increases its mechanical stability and ease of handling, for example, non-permeable impregnation by a silica aerogel slurry or silica sol. The strengthening of interparticle necks (Figure 9), the most vulnerable regions within the aerogel structural network, is responsible for the increase in mechanical characteristics [8]. Without excessive sintering, the hydrolyzed silane sol could create Si–O–Si cross-linking networks by cross-linking with the fiber surfaces. More significantly, cross-linking agents, such as Tetraethyl orthosilicate (TEOS), Methyltrimethoxysilane(MTMS), and Dimethoxydimethylsilane(DMDMS)—hydrolyzed silane sols with various hydroxyl groups—have been studied (Figure 9). According to the mechanical property measurement, SNF/TEOS or DMDMS aerogels displayed 10% and 2% plastic deformation loading of 50% compressive strain after the fifth cycle, respectively; however, SNF/MTMS aerogels failed to show plastic deformation. This indicates that either the rate of cross-linking or the flexibility of the cross-linkers determine the degree of elastic durability [43].

## 6. Mechanism of the Network in the Gel

**Scheme 1:**
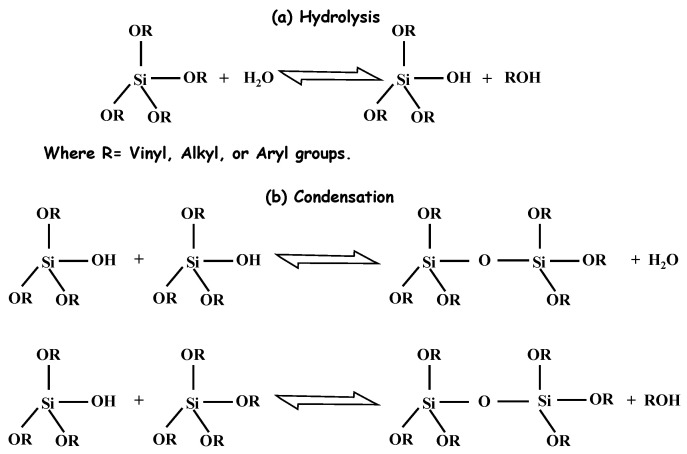
Typical schematic representation of silica gel network formation through sol–gel method.

The aerogel network is established through two primary processes: the breakdown of the precursor (hydrolysis) and the subsequent condensation of the gel. These two phases are regarded as important for the development of the network within the aerogel. The synthesis of silica via the sol–gel process involves three consecutive steps, as outlined in reactions (a)–(b). The first stage entails the hydrolysis of silica alkoxy (Si–OR) groups, leading to the production of silanols (Si–OH) (reaction (a). The second phase, oxolation, initiates when two silica hydroxy groups undergo a reaction (reaction (b)), succeeded by the third stage, alkoxolation, which transpires when a silica hydroxy group engages with a silica alkoxy group. Sequential condensations yield siloxane units (Si–O–Si bonds) and extend the polymer chain, finally creating a network node. Covalent bonds produce a stable three-dimensional matrix structure. The hydrolysis of tetra-functional and trifunctional silicon alkoxides is conducted under suitable circumstances. A catalyst is required for silicon alkoxides to facilitate hydrolysis and condensation reactions. Since silicon alkoxides and water are incompatible, alcohol must be added. Scheme 1 shows the hydrolysis and reaction of condensation [44,45,46].

**Scheme 2:**
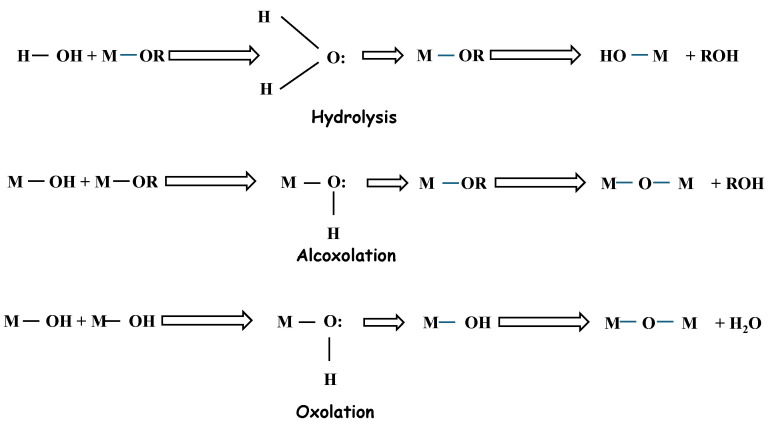
The primary mechanism of the network formation in the aerogel with metal.

First, for a precursor like metal oxide, the hydrolysis of the precursor along with nucleophilic substitution with the help of the hydroxyl group occurs. The second step involves the removal of water molecules (H_2_O) with cross-linking in groups like M-OH (M = metal) or the removal of alcohol combined with condensation between groups like M-OR and M-OH. The formation of a mechanically stable network arrangement occurs only through the reaction of cross-linking and the interior changes in the structure, or both processes continue to alter. The primary mechanism of the network formation in the aerogel with metal can be represented as follows:

Alkoxide ligands readily protonate when present in an acidic solution. The poly-condensation reaction also known as gelation (in macroscopic form) leads to the formation of a 3D network between the bonds with a rigid structure. The aging of the gel also includes polymerization. Aging causes uninfluenced MOH and MOR monomers to compress, strengthening and increasing the gel network’s interaction. Scheme 2 depicts the primary mechanism for gel network formation with metal in the aerogel. Syneresis, or the evacuation of moisture through the voids, can follow this, shrinking the gel network. Several substrate porosities may additionally occur in dissolution–precipitation methods, which occupy tiny pores and dissolve tiny elements (coarsening). As a result, the standard size of pores increases and the distance between them decreases.

For example, basic or acid catalysts are commonly used to catalyze the sol–gel chemistry of silica. The hydrolysis of several alkoxy groups can occur based on many factors, such as the ratio of silane to H_2_O. The rate of each hydrolysis process is determined by the consistency of the phase change region, resulting in an effect that is dependent on the respective electron-donating or -eliminating strength of the -OH compared to -OR groups. Consequently, under basic conditions, succeeding hydrolysis processes become faster, while in acidic conditions, they eventually reduce in velocity. According to this, the presence of an acid or a base causes condensation. In each instance, metaloxane or siloxane are formed in the bonds for different metals [7,45,47].

## 7. Fundamentals of Catalysis

### 7.1. Catalysis

Catalysis is an approach where a catalyst is added to a reaction to help it proceed. This reaction creates a new molecule by disassembling, rebuilding, rearranging, and recombining the atoms in the bonding molecule.

#### 7.1.1. Electrocatalyst to Produce Hydrogen

Hydrogen, among the globe’s naturally occurring components, is a versatile power source used in cars, trains, planes, steel, and ammonia production. Investigating the development of hydrogen is crucial as it represents a highly effective way to substantially minimize emissions. Hydrogen’s economic growth is limited. Hydrogen has been recognised as a practical green fuel for more than a decade. Discovering eco-friendly hydrogen manufacturing systems or materials is a top priority [48,49]. We need to produce electrocatalysts to enhance the reaction rate alongside a decrease in the consumption of electrical energy. Presently, standard Pt-based materials and Ru/Ir-based oxides are in focus as an efficient HER and OER catalyst, respectively. Therefore, substantial research has been carried out to fabricate multifarious non-precious alternatives with outstanding activity and stability, e.g., metals/alloys, their corresponding oxides/phosphides/sulfides/selenides/nitrides/carbides (TMCs), metal-free carbon, single atoms, and various composites [24,25,26,27,28,29,30,31]. These materials have been widely reported and hold high promise for use in electrochemical water splitting. The materials that accelerate electrochemical processes at the surfaces of electrodes or solid–liquid interfaces are known as electrocatalysts. The electrocatalyst’s wide range of applications depends on its cost and efficiency. The renewable energy performance of the system for electrolysis is increased by applying electrocatalysts. The development of long-term and ecologically friendly systems for the electrochemical conversion and storage of energy processes involves the use of electrocatalysts [50].

#### 7.1.2. Water Electrolysis

This method is the oldest to produce hydrogen, originating in the 19th century, which usually involves two electrodes connected by an electrical power supply (DC current) kept inside water. A separator acts as a barrier between the two sections of the chamber. The electric current is used to split the water molecule into hydrogen (H_2_), collected at the cathode, and oxygen (O_2_), collected at the anode inside a unit or system called an electrolyzer. These produced gases of hydrogen and oxygen are collected via gas receivers. The rate of the reaction can be elevated by the utilization of an electrocatalyst, and electrolysis efficacy is increased by putting salt and base or an acid as an electrolyte into the electrolyte solution [51]. The efficacy of hydrogen production varies amongst various kinds of electrolyzers. For example, the alkaline electrolyzer, which requires an alkaline electrolyte (often potassium hydroxide) to function, is among the very first and most well-established techniques [52].

#### 7.1.3. The Basic Concept of Electrochemical Water Splitting

The process of electrochemical water splitting is responsible for the formation of pure hydrogen and oxygen. An electrolyzer consists of an anode, cathode, and aqueous electrolyzer. The splitting of the water takes place after passing an electric current along the electrode in the electrolyte solution. The reaction at the cathode shows the production of H_2_ gas, as the transfer of the electron has occurred in the negatively charged area of the electrolyte. To conserve the electrochemical balance, the ions traverse the electrolyte at the anode, where oxygen (O_2_) is formed after the elimination of electrons. Equation (1) can also be derived from the alkaline and acidic medium. The water electrolysis reaction, free from any collateral reactions, is given below:

(1) H2O→H2+O221

The reaction can be differentiated in two halves, which are the hydrogen evolution reaction (HER) and the oxygen evolution reaction (OER), both of which are dependent upon the characteristic of the active ions present in the reaction. In the acidic solution, protons are active, whereas in the alkaline solution, hydroxides are active ions. The water-splitting reactions depending upon electrolyte solutions are given below:

At cathode

In acidic solution

$$2H^{+}+2e^{-}\;\to {\;H}_{2}$$

In alkaline or neutral solution

$$2H_{2}O+2e^{-}\;\to {\;H}_{2}+2{\mathrm{OH}}^{-}$$

At anode

In acidic solution

2H2O→O221+2H++2e−

In alkaline or neutral solution

2H2O →O221+H2O+2e−

The HER is more active in the acidic medium, while the OER is inactive because components of the electrolyzer are in an acidic medium, resulting in serious corrosion in OER electrocatalysts; alkaline electrolytes are preferred for the water splitting in the process of OER [53].

#### 7.1.4. Hydrogen Evolution Reaction (HER)

The process is widely used for metal-based catalyst transitions, at the cathode, transporting two electrons using a single catalytic intermediate. The catalytic activity and the HER mechanism are strongly reliant on the surface of the catalyst, its interactions with water and reaction intermediates, and the produced molecular hydrogen. The transfer of the two-electron mechanism of HER for catalysts in an alkaline medium depends upon the adsorbed hydrogen ions over the surface of the electrode, water with O-H cleavage bond, generation of the H-H bond, and, lastly, on the discharge of molecular H_2_.

Generally, there are two phases in the HER process in both acidic and alkaline media: (a) Volmer–Heyrovsky and (b) Volmer–Tafel. The hydrogen in acidic electrolytes undergoes an evolution through either the Tafel or Heyrovsky steps following the Volmer reaction, contingent upon the H* surface coverage. The water molecules in the alkaline electrolyte serve as the proton source.

Step 1: Adsorption of electrochemical hydrogen

In the acidic medium, one hydrated proton (H_3_O^+^) first interacts with one electron in acidic electrolytes, forming a chemical bond with the catalyst * through the Volmer process. Two adsorbed H*s then combine to generate H_2_ through the Heyrovsky or Tafel pathway, which undergoes successive desorption. While the Heyrovsky reaction involves mixing the produced H* with a hydrated proton, the Tafel reaction immediately combines two H*s to yield H_2_. After that, the resulting entity forms H_2_ by obtaining an electron from the catalyst surface. Water adsorption constitutes the initial phase of the HER in alkaline environments and occurs at a significantly slower rate than H_3_O^+^ adsorption on the surface, which accounts for the suboptimal kinetics of HER in alkaline conditions [54].

In acidic conditions:

$$H_{3}O^{+}+\;*\;+{\;e}^{-}\;\to H^{*}+H_{2}O$$

**Figure 10:**
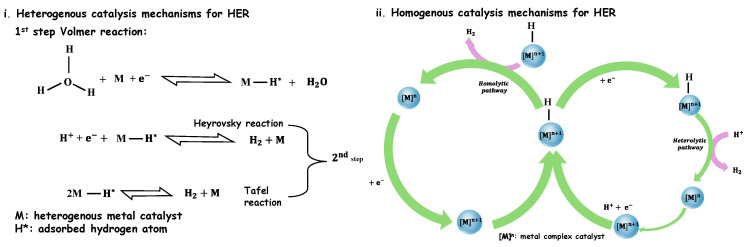
Schematic representation of HER reaction pathways.

In the alkaline medium for H_3_O^+^ or H_2_O reduction, the process of forming adsorbed H* and OH^−^ before H* adsorption is more complex, as the catalyst needs to break the H-O-H bond. Essentially, the affinity between the catalyst surface and the reaction intermediate determines the rate-limiting phase in the electrocatalytic HER process. A weak connection of the catalyst to adsorbed H* will cause adsorption to control the HER process. On the other hand, the desorption process is the step that determines the rate if the bond between the catalyst and the adsorbed H* is excessively strong [55]. Figure 10 provides a schematic representation of the HER pathway.

In alkaline conditions:

$$H_{2}O+\;*\;+{\;e}^{\_}\to H^{+}+{\mathrm{OH}}^{-}$$

Step 2: Tafel reaction

$$H^{*}+H^{*}\;\to H_{2}$$

Heyrovsky reaction

In acidic medium

$$H_{3}O^{+}+{\;H}^{*}+{\;e}^{-}\;\to H_{2}+H_{2}O$$

In alkaline medium

$$H_{3}O^{+}+{\;H}^{*}+{\;e}^{-}\;\to H_{2}+{\mathrm{OH}}^{-}$$

where the catalyst surface is * and the adsorbed hydrogen is H*.

The traditional guidelines for designing catalysts frequently depend on the use of Gibbs free energy (ΔGH), a commonly used metric to evaluate the catalytic performance of a catalyst. The strength of hydrogen binding to the catalyst surface is shown by the ΔGH* value. When ΔGH* is less than zero, it indicates that active hydrogen (H*) is more advantageous to adsorb onto the catalyst’s surface than to desorb. In contrast, a positive value (ΔGH* > 0) indicates that adsorption is not advantageous, but the desorption of active hydrogen from the catalyst’s surface is. A catalyst that exhibits favorable H* adsorption and desorption at the same time would preferably have a ΔGH* near to zero, which could result in optimal kinetics and activation for the hydrogen evolution reaction [56].

#### 7.1.5. Oxygen Evolution Reaction (OER)

**Figure 11:**
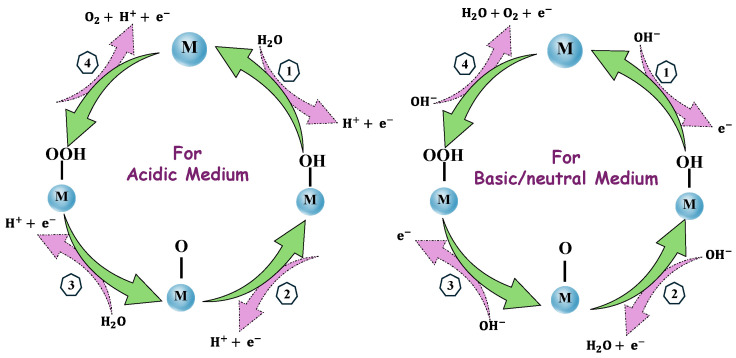
Schematic representation of OER reaction pathways. (The numbers in the pathway represent the reaction in ordered form from 1–4. The different colour arrows, i.e., green represents the reaction occurring for active catalytic sites, whereas the pink colour arrows explains about the electron/proton transfer in the pathways).

Comprehensive research has been conducted on the promotion of OER by aerogels, transition metal oxides, and hydroxides. In comparison to HER mechanisms, OER pathways and mechanisms are comparatively more intricate. The mechanism of OER is dependent on the electrolyte’s pH and the electrocatalyst. The OER mechanism varies for various materials with distinct surface patterns, and oxygen is often developed from the material surface rather than any metal. There are three main intermediate principles for the mechanism of OER: *OH, *OOH, and H* [57]. Even though having the same composition, aerogel can exhibit distinct electrokinetic profiles because of variations in the interconnected layers’ thickness, structure, and techniques of manufacture [58]. The results for the kinetic show sluggish mechanisms because HER is faster than OER, where energy bonding of hydrogen is too fragile or firm throughout the process. Thus, a highly valued overpotential is needed for the OER mechanism [57]. The first step in the OER mechanism includes the separation of the water molecule into hydrogen and oxygen in an acidic medium. Despite this, the following general mechanism for the OER has been proposed and is shown in Figure 11:

In acidic medium:

$$H_{2}O+\;*\;\to *\mathrm{OH}+H^{+}+e^{-}$$

$$*\mathrm{OH}\to *O+H^{+}+e^{-}$$

$$H_{2}O+*O\to *\mathrm{OOH}+H^{+}+e^{-}$$

$$*OOH\;\to \;O_{2}\left(g\right)+H^{+}+e^{-}$$

In alkaline or neutral medium:

$${\mathrm{OH}}^{-}+*\to \mathrm{OH}*+e^{-}$$

$${\mathrm{OH}}^{-}+*\mathrm{OH}\to *O+H_{2}O+e^{-}$$

$${\mathrm{OH}}^{-}+*O\to *\mathrm{OOH}+e^{-}$$

$${\mathrm{OH}}^{-}+*\mathrm{OOH}\to *+O_{2}+H_{2}O+e^{-}$$

The hydroxyl radicals initially adsorb on the catalyst surface through the OER process, forming *OH. The *OH then undergoes the removal of a proton and an electron to produce *O. The *OOH intermediate is subsequently produced by the nucleophilic assault of OH^−^ on the *O site. Then, the oxygen molecule is released, and the catalytic site is recovered by completing the proton–electron transfer process [59].

The thermodynamic Gibbs free energy for an ideal catalyst is 1.229 V at each step. The phase with the largest ΔG in the OER process yields the theoretical overpotential. 

For the four-step process, the magnitude of ΔG_OER_ is computed as ΔG_OER_ from Equation (2).

(2) $$\Delta G_{\mathrm{OER}}=\max\;(\Delta G_{1},\Delta G_{2},\Delta G_{3},∆G_{4})$$

The varied thicknesses, topologies, and techniques of fabrication of oxides are directed towards distinct OER mechanisms, such as oxygen evolved from the surface of metal oxides [60].

## 8. Aerogel as Catalyst

Aerogels’ large surface area makes them useful for a variety of tasks, including spill cleanup and organic solvents as a chemical absorber. It also has the nice attribute of having intriguing use as an interface or catalyst. Aerogels support both gaseous and liquid phase reactions during heterogeneous catalysis. There is extremely appealing potential for catalysis due to the high porosity and extremely large surface area per unit mass.

Sol–gels exhibit qualities that make them suitable materials for catalytic applications; studies on this topic have been published. There are two types of catalytic reactions: redox and acid–base. As with all oxides, distinct Brönsted or Lewis acid site types may coexist in the latter scenario. One traditional method involves replacing the host oxide with dopant cations that have a different valence state to add more or stronger acid sites. For instance, in SiO_2_, trivalent Al^3+^ cations can be used in place of tetravalent Si^4+^ cations to utilize a tetrahedral Si site [61].

### 8.1. Aerogel as an Electrocatalyst

The application of novel hybrid techniques has made electrocatalysis an essential field in electrochemistry. The primary variables that impact the characteristics of electrocatalyst atom rearrangements include the influence of surface roughness, identification of catalytic center sites, phase transitions, and on the catalytic surface that occur during electrochemical processes [62,63].

It is still difficult to develop an appropriate electrocatalyst with the right balance of activity, stability, and selectivity to carry out the electrochemical process. Although the electrocatalytic approach is now thought to be a promising study technique for H_2_ evolution, it has limitations, including low product selection and Faraday efficiency (FE). Thus, for electrocatalysts for electrochemical production, the development of efficient catalysts that have decreased overpotential, high FE, and selectivity for products is essential. By changing the frequency where oxidation and reduction reactions take place, the electrocatalysts enable specific chemical interactions at the electrode surface [22]. A fundamental benefit of aerogels in all situations is the relatively high specific surface area, which enables a wide range of active locations per gram of substance [61].

#### 8.1.1. Silica Aerogel with Catalyst Hydrogen Generation

The deposition of chemical catalysts in SiO_2_ aerogel enhances their vulnerability to contaminants, thereby elevating the quantity of harmful substances that reach the sites that react for photocatalytic and photoelectrochemical (PEC) removal. Hydrogen emerges as a noteworthy clean energy source, as its use leads to the generation of gas from solely water. Nonetheless, the existing literature on SiO_2_ aerogels as for catalyst loading is quite limited. Moreover, SiO_2_ serves as a charge transfer catalyst that promotes the mobility of photo-generated electrons and holes, consequently improving photocatalytic performance. The research developed by Yu Ju Pin et al., a cobalt-based silica aerogel (Co/SAG) nanocomposite through a straightforward chemical reduction process, serves as a catalyst for hydrogen production from the hydrolysis of NH_3_BH_3_. The nanocomposite showed a hydrogen generation rate that surpassed Co/MCM-41 (cobalt/ordered mesoporous silica) by 41%, underscoring its promise as an inexpensive catalyst for hydrogen production. The presence of smaller (~<5 nm) and uniformly distributed Co nanoparticles is evidenced by TEM analysis [64]. Silica microspheres were created through a one-step atmospheric drying process by Yue, Xian et al., whereas titanium dioxide with silicon dioxide with surface area (400 m^2^/g) (TiO_2_@SiO_2_) composites were developed using liquid phase deposition techniques. The enhanced material demonstrated H_2_ production rates approximately three-times greater than P25 TiO_2_, highlighting its promise for sustainable hydrogen generation [65]. The study from Somakli and Senguel showed the synthesis of silica aerogels using a hydro(solvo)thermal-supported sol–gel technique. After 3-Aminopropyl triethoxysilane (APTES) modification and hydrochloric acid protonation, SiO_2_ and carbon particles (CP) were inserted to create an interpenetrating network. When utilised as a catalyst for NaBH_4_ methanolysis, the resultant CP-APTES-HCl silica aerogel produced 1860 mL min^−1^ g^−1^ of hydrogen at 298 K [66]. The group of S.S. Rayalu prepared platinum (Pt) and ruthenium (Ru) to promote titanium dioxide, which was supported on mesoporous silica. This increased the surface area of 140.6 m^2^/g and visible-light photocatalytic activity in comparison to a mixed-phase TiO_2_ nanomaterial (Degussa P25). Through characterisation techniques, the supported photocatalyst demonstrated a remarkable hydrogen yield of 4791.43 μmol/h/g TiO_2_, which is 30-times greater than that of P25 (161 mmol/h/g TiO_2_), utilising ethanol as a sacrificial donor [67]. The group of Jordi Llorca introduced a novel method to minimize dispersion throughout the production of hydrogen from an ethanol–water mixture, where cobalt talc nanolayers were immobilized within the SiO_2_ aerogel. This approach further ensured a significant mass flow among reactors, catalysts, as well as products [68]. The incorporation of SiO_2_ aerogel serves as a strong structural arrangement, reinforcing the composite structure while offering comprehensive and uninterrupted surfaces that facilitate improved catalytic performance, thereby establishing an excellent stimulating system within the composite.

#### 8.1.2. Factors Affecting Electrocatalysts

There are certain essential factors associated with water splitting, such as the overpotential (*η*), Tafel slope (*b*), current density (*j*), and Faraday efficiency, which can be used to evaluate the electrocatalytic abilities of catalyst materials.

#### 8.1.3. Overpotential

The differences between the reversible cell potential (Er) and the actual cell potential (E) are known as the overpotential. It measures how ineffective electrochemical reactions are. Overpotentials are positive for oxidation reactions and negative for reduction reactions. The activation overpotential occurs at the anode and cathode due to intrinsic activation barriers [69]. Concentration gradients in the vicinity of the electrode contact cause concentration overpotentials. Overpotentials are decreased by high electrochemical activity in electrodes and catalysts. Selecting the right catalyst can lower the activation overpotential. Conditions for electrolysis mean that a lower overpotential is needed for electrolysis in very acidic or alkaline solutions [70,71].

#### 8.1.4. Tafel Slope and Current Density

A HER–OER electrocatalyst that is consecutively affordable, active, and long-lasting for both (HER and OER) in the same electrolyte is the perfect one for full water splitting [72]. Tafel graphs can be used to illustrate how the equilibrium density of current for water splitting depends on the anodic or cathodic overpotentials (*η_a_*, *η_b_*). The linear part of the overpotential (*η* > 0.05 V) is represented by Equation (3):

*η* = *a* + *b*
*log*(_*j*_) (3)

where *a* is the Tafel constant, and *b* is the Tafel slope, which is associated with the reaction mechanism of the electrode.

A facile electron transport with low activation energy is indicated by a high value of *j*. The overpotential is typically logarithmically connected to the exchange current density (*j*_0_) generated by the above equation when *η* = 0. This illustrates the catalysts’ inherent activity in equilibrium conditions. A point for which the electrocatalytic process’s equilibrium potential, or zero potential, meets the corresponding current density on a logarithmic scale is the intersection of the linear, which is extrapolated to acquire the exchange current density. The higher the exchange current density, the more effective the electrocatalyst; the type of electrocatalyst is temperature-dependent, and the intrinsic rate of reaction transfer between the electrode and electrolyte is under equilibrium conditions [60,73,74].

#### 8.1.5. Faraday Efficiency

When electrons are moved effectively during oxidation or reduction reactions in an electrochemical system, this is referred to as Faradaic efficiency [9]. It shows how well electrons from an external circuit propel Faradaic processes, which result in losses in the form of heat or waste. Faradaic loss can arise from electrons or ions taking part in unintended side reactions in both electrolytic and galvanic cells. These losses appear as chemical byproducts or heat. For instance, during the electrolysis of water, some electrons that were meant to produce hydrogen may instead be used to produce hydrogen peroxide, which causes a Faradaic loss. Negative outcomes in cold fusion studies have been mistakenly identified due to this effect’s failure to be taken into consideration.

The usual method for determining Faradaic efficacy is bulk electrolysis. The quantity of substances discovered contrasts with the theoretically predicted quantity after a specific amount of reagent is stoichiometrically transformed into a product. Experimental hydrogen production can be quantified using analytical techniques including gas chromatography and water–gas displacement. Within an electrochemical system, there are various ways in which energy can be lost. One such way is through Faradaic loss. Overpotential is an additional component that explains the discrepancy between the real and theoretical electrode voltages required to drive the reaction at the intended rate. In general, energy efficiency is impacted, since even a 100% Faradaic-efficient rechargeable battery needs to be charged at a voltage higher than it generates when discharged [75].

#### 8.1.6. Turnover Frequency (TOF)

The rate at which the electrocatalyst can catalyze the electrochemical reaction at a specific overpotential is indicated by another kinetic parameter called TOF. The quantity of O_2_ or H_2_ gas evolving at every possible catalytic site is what defines it. Because they both have pseudo-first-order kinetics, HER and OER have time-of-flight values (or TOF values). Although TOF is not affected by mass loading, it is heavily reliant on high coverage; that is, it only exhibits a linear relationship when the surface area is less than 100%. The catalyst is better the higher the TOF value.

$$\mathrm{TOF}=\frac{jA}{\alpha F_{n}}$$

where the number of catalyst electrons is denoted as *α* (electron/mol), Faradaic constant is denoted as *F*, the metal atoms covered on the electrode (mol) are denoted as *n*, the surface area of the working electrode is denoted as *A*, and the current density is denoted as *j* [60,70].

#### 8.1.7. Stability

When evaluating an electrocatalyst’s viability for large-scale, commercial operations, stability becomes a critical factor to consider, since it affects the catalyst’s performance over a prolonged amount of time. Two methods are used to study the stability of HER and OER by cycling the catalyst throughout the potential window: extended potentiostatic or galvanostatic (the process of electrolysis evaluations) and cyclic voltammetry (CV) or linear sweep voltammogram (LSV) at more substantial scan rates, referred to as the accelerated degradation test. The processes of HER and OER have different stabilities for catalytic material, which is dependent on the number of cycles performed. In the case of HER, the polarization curve begins at 0 V vs. NHE as thousands of cycles are being carried out. The literature for the OER process shows that accelerated degradation is not possible with thousands of cycles as it is rare, but it can be possible within 250–1000 cycles, with the OER catalyst showing maximum stability. There are some factors, like the catalyst fixation, the electrode that is used, and the substrate, that are responsible for the stabilization of the electrocatalyst, which is under process [60].

#### 8.1.8. Activity Descriptors for Silica-Based Aerogels in Hydrogen Generation via Water Splitting

The creation and refinement of effective and stable electrocatalysts aimed at minimizing electricity usage are increasingly crucial for enhancing the parameters of green hydrogen technologies. As discussed, the combination of structural and design parameters of the final catalyst device should be optimized for each device separately to obtain maximum catalytic efficiency [76].

Establishing activity descriptors that link the structural and chemical characteristics of silica-based aerogels to catalytic or photocatalytic effectiveness is essential to enable their systematic design for hydrogen generation. These metrics serve as prognostic guides to improve aerogel performance in water-splitting processes. These descriptors serve as predictive parameters for optimizing aerogel performance in water-splitting reactions.

I. Surface Area and Porosity: The maximum active site exposure enables reactant diffusion in the silica aerogels because of high surface area and linked porosity. The percentage of total porosity, pore size distribution, and specific surface area is calculated using Brunauer–Emmett–Teller (BET) analysis.
II. Hydrophilicity and Surface Functionalization: The interactions of water and catalytic activity change with the degree of surface hydroxylation or modification with functional groups (e.g., -OH, -NH_2_) and can be identified by the contact angle, zeta potential, X-ray photoelectron spectroscopy (XPS)-based hydroxyl group measurement.
III. Electronic Band Structure: The silica aerogel has specific band gap energy that is responsible for how much light is adsorbed by the aerogel in the photocatalytic water splitting. The analysis in conduction and valence band edges is investigated using Mott–Schottky analysis as well as bandgap energy characterized by UV-visible diffuse reflectance spectroscopy (UV-Vis DRS).
IV. Charge Carrier Dynamics and Photocatalytic Efficiency: The effective separation and transfer of photogenerated charge carriers determine hydrogen generation as well as photocatalytic efficiency. Analysis for evaluating recombination includes photoluminescence (PL) intensity; for charge lifetime, transient absorption spectroscopy; for measuring charge transfer resistance, electrochemical impedance spectroscopy (EIS).
V. Stability and Reusability: The aerogels’ long-term stability and recyclability are responsible for the aqueous and oxidative systems, which guarantee useful applicability. Structural integrity via TEM/SEM can be assessed after the reaction, and weight loss can be calculated through TGA measurement.
VI. Metal/Oxide Doping Effects: Metal/oxide doping dopants, like TiO_2_ [77], Fe [78], Co [79], and MoS_2_ [80], can increase catalytic activity and enable charge separation. The elemental composition examined using XPS and EDS, oxidation state calculated by Operando X-ray absorption near-edge structure (XANES), extended X-ray absorption fine structure (EXAFS), and local atomic structure described by Raman spectroscopy are represented by characterizations.

## 9. Aerogel with Plasma Treatment

The plasma process consists of sputtering effects caused by ion bombardment along with chemical reactions between radicals and surface bonds produced in the discharge volume. The plasma medium is made of radical particles, electrons, ions, UV radiation, and soft X-rays. Solid surface pollutants or liquid coatings may be treated by plasma by changing into volatile reaction products that are pumped out of the system. The ability of plasma to penetrate is, certainly, a crucial feature. One may argue that plasma treatment is an effective technique for cleansing applications because of its advantages in terms of technology, the environment, and cost. The characteristics of the material’s surface, gas species, radiofrequency (RF) power, and gas pressure all affect the performance of the process [81,82]. Table 4 gives a comparison for silica material, reactive and non-reactive gases, and their advantages.

SiO_2_ aerogel is a low-k material that shows promising use in intermetal dielectric (IMD) applications because of its flexibility and low dielectric constant. A sol–gel polymerization process followed by a drying process creates highly porous SiO_2_ aerogel film by preventing pore structure collapse. After drying, surface chemical species like Si-OR (R: alkyl) and Si-OH groups are still present because of the incomplete polycondensation reaction. Consequently, numerous methods have been suggested for implementing it in IMD. The highly polarizable species described were shown to be responsible for the observed loss of electrical characteristics, such as leakage current density and dielectric constant [83,84].

**Table 4:** Comparison table for silica material, reactive and non-reactive gases and their advantages. (↑ = high, ↓ = low).

Sr. No.	Material	Reactive Gases	Non-Reactive Gases	Advantages	Reference
1	SiO_2_ film	O_2_, H_2_, N_2_	He, Ar	Dielectric current ↑ Leakage current ↑ Thickness (O_2_ > N_2_ > Ar > He ≅H_2_)	[82]
2	SiO_2_ film	O_2_ (Si/O/C)	-	Thickness of film ↓ (900–700 ± 10 nm)	[85]
3	Mesoporous silica thin film	H_2_, O_2_	-	Leakage current ↑ H_2_ = 3.6 ×10^−6^ A/cm^2^ O_2 =_ 1.17 ×10^−4^ A/cm^2^	[83]
4	Amorphous Silica Nanoparticles	N_2_, O_2_	-	Specific surface area = 124 − 420 m^2^/g Low Thermal Conductivity = 0.00014 W/mK	[86]
5	Nanoporous Silica thin films	O_2_, H_2_, NH_3_	-	Dielectric constant density below 1 × 10^−7^ A/cm^2^C	[87]

## 10. Silica Aerogel as Open Cell

Huijin Wu et al. synthesized light and ultra-light silica aerogel foam concrete through an advanced process. The method for producing lightweight aerogel with equal volume replacement and drying was performed at atmospheric temperature. The synthesis was carried out in the following stages: preparation mixing and blending, foaming, curing and shaping. The as-prepared light aerogel is replaced by ultra-light aerogel, as it shows low density with reductions of 24% and 63% porosity. The aerogel shows good performance for thermal insulation and can be used for low-carbon buildings [88].

Kayal et al. reported Ag NPs to be anchored to the functionalized SBA-15 surface. The Ag-containing nanomaterial performs better when it comes to OER due to the substantial mesoporous support’s particular surface area and strong electron capacity of the Ag NPs to donate. Additionally, the uniform distribution of the tiny, spherical silver nanoparticles can boost the active site density at the surface of the material, leading to more effective activity involving electrocatalysis [89].

Nawaz et al. showed a comparison of silica-aerogel-based adsorbents to other commercially available desiccants, including activated carbon. These investigations demonstrate that the former has a lower temperature of regeneration and a better adsorption capacity. The viability of these materials for sorption systems cannot be determined despite their encouraging qualities until a dynamic examination of the silica aerogel-coated substrate’s adsorption and desorption performance under practical working conditions has been conducted. The mass diffusivity influences the adsorption rate, and the adsorption isotherm determines the capacity of a porous adsorbent material in the adsorption of an adsorbate gas [90].

Hou W.-L. et al. reported the synthesis of silica foams having an ultra-large specific area (1344.23–1733.28 m^2^/g), with a structure of hollow mesoporous silica spheres. The method of particle-stabilized foaming was used to prepare 3D network hollow mesoporous spheres. They obtained ceramic foam with high porosity and macropore structures in regular form. From SEM, it was observed that the cell wall of the hollow sphere was 200–600 nm in range, showing apparent open pores [91].

## 11. Aerogel as Adsorbent for Dye

The as-prepared mesoporous silica with a large pore size, stated as mesostructured cellular foam (MCF), was reinforced for the laccase immobilization with the help of an adsorption cross-linking process by Min Zhao et al. The decolorization of dyes Indigo and Alizarin red was studied for enzymes like ABTS with the immobilized laccase. The rate of discoloration was low in the absence of ABTS. But, as the ABTS was added, the rate of discoloration was increased by 73% and 97%, respectively. Here, the ABTS acts as the mediator for the dye’s discoloration. Physical absorption along with cross-linking enhanced the results for the discoloration of dyes Indigo and Alizarin red [92].

Zhou Zhai and Yuan Dong used a hydrothermal method for the synthesis of mesocellular silica foam. They calculated the pore size of the mesocellular silica foam before and after the adsorption of the methylene blue, where the pore diameter and pore volume observed were 9.34 nm and 1.36 cm^3^ g^−1^, which decreased with values of 8.03 nm and 0.67 cm^3^ g^−1^ after the adsorption of methylene blue. The surface of the foam silica with a mesocellular structure with methylene blue was 399 m^2^ g^−1^, and in its absence, it was 585 m^2^ g^−1^. The results showed a decrease in the total pore volume, average pore size, and specific surface area because the holes of mesocellular silica foam were partly filled with methylene blue after its adsorption [93].

Qun liu et al. reported a novel method for the synthesis of silica aerogel from precursor polysiloxane E-40, used as industrial reagent polysiloxane and less expensive than other precursors like TEOS and TMOS. The precursor has a larger amount of silica than TEOS, so it is a good means to obtain organic silicon1. The synthesis procedure has three stages: sol–gel, altered replacement of organic solvent, and ambient drying. The pore diameter of the three aerogels is 19.5 nm for ambient pressure drying, 22.3 nm for supercritical drying, and, for the hydrophilic aerogel (HSA), it is 28.8 nm. The removal percentage of dyes for HSA as an adsorbent is 98, which fitted the Bore–Zwikker model that reveals adsorption to be heterogeneous [94]. Table 5 shows a comparison table for different silica materials, pore diameters and volume, dye adsorbed, adsorption capacity of the silica, adsorption isotherm, and process, which is represented as follows.

## 12. Conclusions and Outlook

The distinctive properties and varied chemical structures define aerogel as an interesting category of matter. Aerogel has been researched for approximately 80 years, and its various types, uses, and manufacturing methods have adapted. Aerogels represent innovative materials characterized by their transparent three-dimensional nanostructures. The enhancement of aerogel surfaces, both physically and chemically, to enhance the adsorption process appears to hold significant promise for foreseeable breakthroughs. Aerogel foams demonstrated evidently reduced bulk density, increased porosity, and diminished thermal conductivity relative to conventional aerogel variants. Their attractiveness lies in their ability to withstand high temperatures, exhibit poor thermal conductivity, and maintain a small refractive index. The properties of aerogels enable numerous uses, such as adsorption, separation, and thermal insulation. The electrical characteristics of aerogels are being utilized in the construction of wearable gadgets, energy storage systems, and supercapacitors. Silica-based aerogels have attracted significant interest lately among the different varieties of aerogels.

In recent years, the advancement of electro- and photocatalysts has acquired significant attention in green energy storage and conversion technologies. Comprehensive studies on electrocatalysts for hydrogen evolution processes are needed due to the global energy transition and sustainable development problems. Our findings suggest the following electrocatalyst research directions for this reaction: (a) Advances in costly metal catalysts. Platinum metal catalysts operate well, but their high cost and rarity limit their use. Enhancing studies on novel, cost-effective metal catalysts is crucial to develop durable, high-performing materials. (b) Thorough promoter modification research and improvement. Due to catalyst problems and limits, ongoing research must study and improve catalyst variation strategies. By carefully evaluating catalyst assembly, effectiveness, and response functions, we may improve their strength management. (c) Advanced catalytic mechanism research. Advances in characterization and computational chemistry require prioritizing catalytic mechanism research.

The key findings suggest that while synthesis technology has been revolutionized, it has also raised issues related to which silica material has the properties to act as a catalyst for hydrogen production with water splitting and the adsorption of dyes as an adsorbent. Additional research will be undertaken to regulate the impacts of the gel formation temperature and delivered at atmospheric pressure. Nonetheless, aerogel currently presents two additional challenges. The primary concern is the expense, which remains significantly greater than that of traditional insulation materials, and the secondary issue is the dust generated during production, which is exceedingly challenging to eliminate. Future projections suggest that the unit price of aerogel will decrease due to potential advancements in materials science and technology. Conversely, the advancement of metallic and multi-metallic aerogel networks, along with their application on the surfaces of electronically performing aerogels (composite material aerogels), is poised to garner significant interest in the upcoming years.

Future research should concentrate on utilizing environmentally friendly materials as sources for aerogel production, both for technical as well as for biomedical applications, manufacturing aerogels that possess the desired mechanical and adsorption characteristics. This strategy is effective in minimizing costs and implementing a novel method for recycling certain materials. Ultimately, it is crucial to acknowledge that the unique structure of aerogels facilitates their surface engineering or functionalization. The essential factor in enhancing water electrolysis efficiency and reducing expenses is the development of high-efficiency, cost-effective electrocatalysts that possess insufficient overpotential and minimal Tafel slope values for the hydrogen evolution reaction (HER) and oxygen evolution reaction (OER). Additional research on this matter will be essential to develop materials capable of eliminating or detecting contaminants and enhancing removal efficiencies.

## Figures and Tables

**Table 1:** Comparative table of synthesis method for aerogel with advantages and disadvantages.

Synthesis	Aerogels	Advantages	Disadvantages
Sol-gel	Inorganic aerogel, Hybrid Aerogels, and composite aerogel	Monoliths formed, Pores size diameter 1 μm, 3D network, cost effective and easy method.	Low crystallinity of gels, amorphous, Hydrolysis and condensation are fast, time consuming.
Self-assembly	Oxide aerogels, Graphene based, metallic aerogel	Structure with large and complex network, Crystalline aerogels obtained	Assembly of aerogel structure is uncontrollable
Emulsion	Inorganic oxide aerogel, Polymer	Spherical shaped micro aerogel can be formed by analysing and refining	Aerogels are usually impure as the elimination of emulsifier is difficult
Template	Graphene, Polymer and Functional Aerogels	Crystalline aerogel with high quality and Intricate formations exhibiting desirable resolutions	Structure of aerogel collapses after the elimination of the template
Epoxy	Oxide aerogel	Control of properties and composition, formation of composite of metal materials is possible	Composed aerogels have amorphous nature, prolonged time period
3D printing	Polymer and hybrid aerogels	Macroporous structure, design of intricate structures is possible	Printing procedures, expensive, solution viscosity maintenance, post-processing (chemical and/or heat) needed to give mechanical strength and structural integrity

**Table 2:** The properties of open-cell and closed-cell foam.

Sr. No.	Property	Open Cell Foam	Closed Cell Foam
1.	Density	Lightweight	High Density
2.	Barrier	Air	Air and Moisture
3.	Cost	Less Expensive	Expensive
4.	Flexibility and Durability	Soft	Hard
5.	R value (insulation’s ability to resist)	High	Low

**Table 3:** The property of the aerogel with value and comments.

Sr. No	Property	Value	Comments
1.	Thermal Conductivity	0.017–0.021 (Wm^−1^K^−1^)	High internal surface area Continuous porous geometry
2.	Bulk Density	0.003 ± 0.500 (g/cm^3^)	Empty spaces are filled by air
3.	Inner Surface Area	100–1600 (m^2^g^−1^)	High porosity
4.	Porosity	80–99.8 (%)	High
5.	Mean Pore diameter	20–150 (nm)	Nanoscale dimension
6.	Refraction Index	1.0–1.05	Very low
7.	Thermal tolerance	>500 °C	At first shrinkage occurs from 500 °C, increasing with an increase in temperature
8.	Modulus of Elasticity	0.002–100 (MPa)	Low
9.	Dielectric Constant	~1.1	Very low
10.	Sound velocity	20–800 (ms^−1^)	Low

**Table 5:** Comparison table for silica material, pore diameter and volume, dye adsorbed, adsorption capacity, adsorption isotherm and process.

Sr. No.	Material	Synthesis	Pore Diameter (nm)	Pore Volume (cm^3^ g^−1^)	Dye Adsorbed	Dye Adsorbed	Adsorption Isotherm and Process	Reference
1	Hierarchically porous silica micro-foam with sulfonic acid (SO_3_H-HSM)	Sol–gel	13.78	-	Rhodamine B, Fuchsin basic, Neutral red	Rhodamine B, Fuchsin basic, Neutral red	Freundlich Isotherm	[95]
2	Mesocellular silica foam	Hydrothermal	9	1.36	Methylene Blue	Methylene Blue	Freundlich and quasi second order	[93]
3	Mesoporous silica (MCM-41)	Hydrothermal	2.4	0.733	Toluidine blue O	Toluidine blue O	Freundlich and quasi second order	[21]
4	Mesocellular silica foam	Hydrothermal	13.44	-	Crystal violet	Crystal violet	Freundlich and quasi second order	[20]

## Data Availability

No new data were created or analyzed during this study. Data sharing is not applicable.
